# Proteome-Wide Mendelian Randomization Identifies Causal Proteins for Multiple Sclerosis Susceptibility and Severity

**DOI:** 10.3390/genes17070810

**Published:** 2026-07-16

**Authors:** Ke Chen, Qin Zou, Yuan Yao

**Affiliations:** 1 Department of Neurology, West China School of Public Health and West China Fourth Hospital, Sichuan University, Chengdu 610041, China; 2 Department of Cardiology, West China School of Public Health and West China Fourth Hospital, Sichuan University, Chengdu 610041, China

**Keywords:** multiple sclerosis, plasma proteomics, Mendelian randomization, pQTL

## Abstract

**Background**: Despite significant advances in identifying genetic risk factors for multiple sclerosis (MS), the causal involvement of circulating proteins in disease development and severity remains largely unexplored. Understanding these protein-mediated pathways could reveal novel therapeutic opportunities for this complex neurological disorder. **Methods**: We performed a proteome-wide Mendelian randomization study integrating data from four large-scale protein quantitative trait loci studies to investigate causal relationships between plasma proteins and both MS susceptibility and severity. **Results**: Our analysis revealed four plasma proteins with significant causal effects on MS susceptibility: ALPI and MXRA8 were associated with increased risk, while IDUA and NID2 showed protective effects. For disease severity, MANSC1 and LYVE1 were associated with worse disability, whereas CBR1 demonstrated protective effects. **Conclusions**: Our study provides genetic evidence supporting associations between specific plasma proteins and MS susceptibility and severity, implicating biological pathways related to gut–brain axis signaling, extracellular matrix remodeling, and oxidative stress. These findings improve our understanding of MS biology and identify candidate proteins for future functional validation, therapeutic investigation, and biomarker research.

## 1. Introduction

Multiple sclerosis (MS) is a debilitating neurological disease characterized by immune-mediated attack on central nervous system myelin, leading to progressive axonal degeneration and disability. The disease’s complex pathophysiology arises from dynamic interactions between genetic predisposition, environmental triggers, and maladaptive immune responses [1]. While current disease-modifying therapies effectively target peripheral immune mechanisms in relapsing-remitting MS, they demonstrate limited efficacy in progressive forms where compartmentalized CNS inflammation and neurodegeneration dominate [2]. This highlights fundamental gaps in our understanding of MS pathogenesis, particularly the protein mediators that translate genetic risk into biological mechanisms.

The pathogenesis of MS arises from complex interactions between genetic susceptibility and dysregulated immune responses, where circulating proteins likely serve as critical mediators [3]. Previous case–control studies have identified a number of plasma protein biomarkers associated with MS [4,5]. However, whether these proteins are mere bystanders or causal drivers of MS remains unclear. Observational studies face major limitations, including confounding and reverse causation, making it difficult to distinguish true disease mechanisms from secondary effects. The advent of large-scale proteomic and genomic studies has enabled the identification of protein quantitative trait loci (pQTLs), genetic variants that reliably influence plasma protein abundance. These pQTLs serve as ideal instruments for Mendelian randomization (MR) analyses, an innovative approach that leverages the random allocation of genetic variants during gamete formation to emulate randomized controlled trials, thereby overcoming major limitations of observational epidemiology [6]. By employing genetic variants as unbiased proxies for protein abundance, MR offers a robust framework for establishing causal relationships between circulating proteins and disease mechanisms. Although previous research has identified protein biomarkers associated with MS, a systematic investigation of their causal roles in MS pathogenesis using this genetic approach remains lacking.

In this study, we performed proteome-wide MR meta-analysis to systematically investigate causal relationships between plasma proteins and MS susceptibility and severity. By harmonizing genetic data from four large-scale pQTL studies with MS genome-wide association results, we aim to identify putatively causal protein biomarkers and prioritize the most promising candidates for functional validation and therapeutic development.

## 2. Methods

### 2.1. Datasets

We analyzed two primary outcomes: (1) MS susceptibility using summary statistics from the largest European-ancestry GWAS (47,429 cases; 68,374 controls) [7], and (2) disease severity using age-related MS severity (ARMSS) scores (*N* = 12,584) [8]. For disease severity, neurological disability was measured using the expanded disability status scale (EDSS), an ordinal numerical scale that increases as neurodegeneration progresses. To control for the effects of ageing, individual EDSS measures were converted to ARMSS score by ranking disability within age-specific strata.

Summary statistics for pQTL were obtained from four GWAS: the first involving 2923 proteins from 54,219 UK Biobank participants with the Olink Explore 3072 platform [9], the second analyzing 4907 aptamers in 35,559 Icelanders with the SomaScan multiplex aptamer assay v4 [10], the third examining 2994 plasma proteins in 3301 individuals of European descent using aptamer-based multiplex protein assay (SOMAscan) from the INTERVAL study [11], and the fourth examining 4775 human plasma proteins assayed by the SomaScan v4 platform among 10,708 individuals of European descent [12]. Both cis-pQTLs and trans-pQTLs reported in the original pQTL studies were considered as candidate instrumental variables. Single nucleotide polymorphisms (SNPs) that passed the genome-wide significance threshold (*p* < 5 × 10^−8^) in the original studies were selected and then clumped based on the linkage disequilibrium structure from the 1000 Genomes Project. Since the sample size of the INTERVAL study was relatively small and fewer significant associations were identified, a more relaxed significance threshold (*p* < 1 × 10^−6^) was utilized to increase the number of eligible instrumental variables. Index SNPs (r^2^ < 0.001 with any other associated SNP within 10 Mb) with the minimum *p* value were retained as instrumental variables. Harmonization was undertaken to rule out strand mismatches and ensure alignment of SNP effect sizes.

### 2.2. Mendelian Randomization Analysis

To evaluate the causal effect of proteins on MS, we performed a two-sample MR analysis. For proteins with multiple instrumental variables (IVs), the random-effects inverse variance weighted (IVW) method was used as the primary analysis; for proteins instrumented by a single SNP, the Wald’s ratio method was applied instead. Subsequently, we conducted a meta-analysis to combine the MR estimates, employing the ‘metafor’ package in R when results from at least two studies were available. To account for multiple testing, Bonferroni correction was performed separately for the MS susceptibility and MS severity analyses according to the number of proteins tested in each analysis. Accordingly, statistical significance was defined as *p* < 1.39 × 10^−5^ (0.05/3611) for MS susceptibility and *p* < 1.35 × 10^−5^ (0.05/3704) for MS severity.

Additionally, we conducted comprehensive sensitivity analyses to assess potential violations of the model assumptions in the MR analysis [13]. These included MR-PRESSO (Mendelian Randomization Pleiotropy RESidual Sum and Outlier) to detect and correct for outlier instrumental variables, Cochran’s Q test to check for heterogeneity across the individual causal effects, and MR-Egger regression to evaluate the directional pleiotropy of instrumental variables. The Steiger directionality test was performed to verify that the instrumental variables explained more variance in the exposure than in the outcome, confirming the hypothesized causal direction. Where MR-PRESSO identified outlier instruments (global test *p* < 0.05), the distortion test was used to assess whether removal of outliers materially altered the causal estimate. In all cases the distortion test was non-significant (*p* > 0.05) and the original IVW estimates were retained. Note that MR-Egger, Cochran’s Q, and MR-PRESSO require a minimum of three instrumental variables and were not applicable for single- or two-IV analyses. The F statistic was calculated to assess the strength of the instrumental variables. Statistical analyses were conducted using the R package TwoSampleMR 0.5.6.

## 3. Results

**Figure 1:**
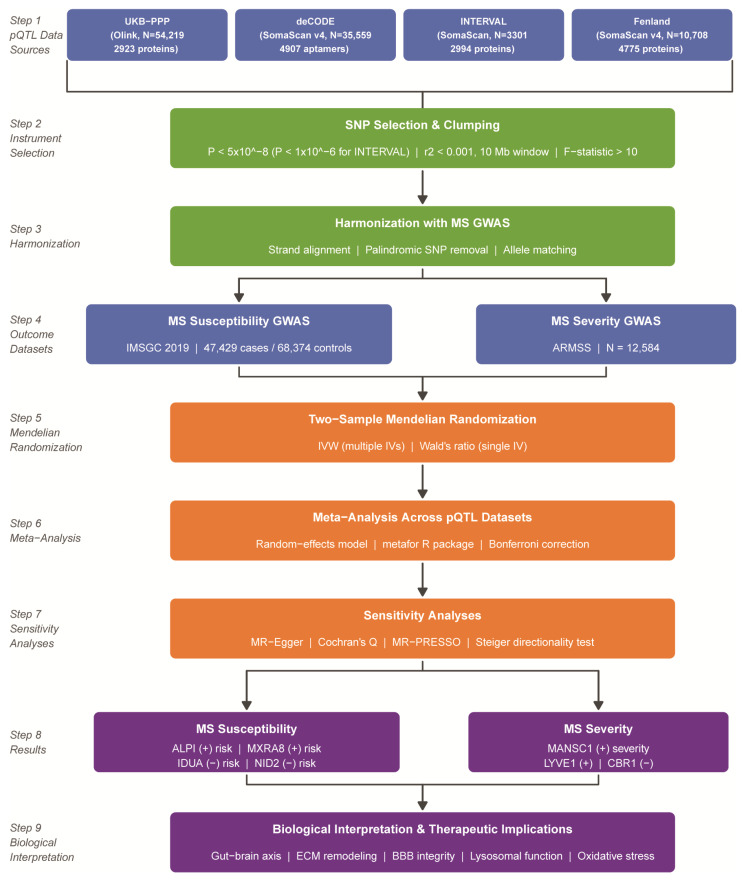
Study design and analytical workflow. Schematic overview of the study design, including pQTL dataset selection, instrumental variable selection and clumping, harmonization of exposure and outcome datasets, Mendelian randomization analysis, meta-analysis across pQTL datasets, sensitivity analyses, and identification of proteins associated with multiple sclerosis susceptibility and severity.

**Figure 2:**
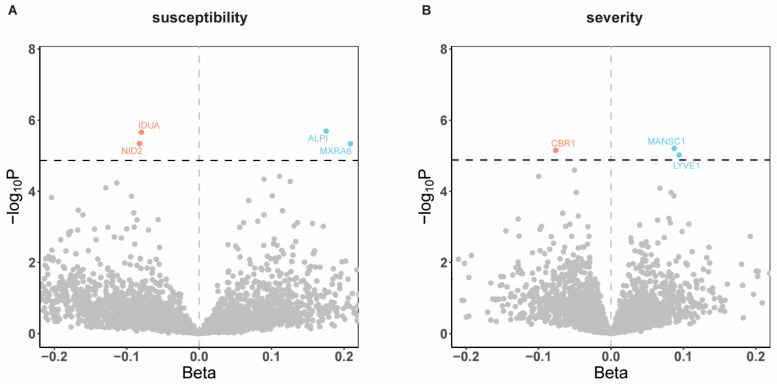
Results from proteome-wide Mendelian randomization meta-analysis. Volcano plots showing the MR effect estimates (beta, *x*-axis) and corresponding *p* values (−log10(*p*), *y*-axis) for the association between genetically predicted plasma protein levels and (**A**) multiple sclerosis susceptibility and (**B**) multiple sclerosis severity. Each dot represents one plasma protein.

**Figure 3:**
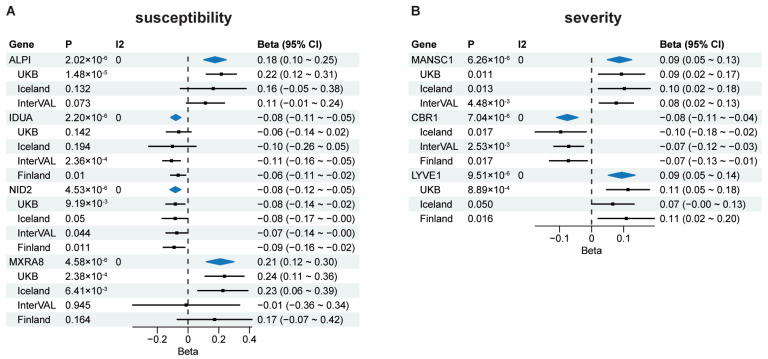
Mendelian randomization estimates for significant proteins across individual pQTL datasets. Forest plots showing the MR effect estimates and 95% confidence intervals for significant protein-multiple sclerosis associations in each pQTL dataset, including (**A**) susceptibility-associated proteins and (**B**) severity-associated proteins. Between-study heterogeneity was assessed using the *I*^2^ statistic.

We conducted protein-wide two-sample MR analysis to assess the causal associations between plasma proteins and MS risk and severity (Figure 1). A total of 3611 and 3704 proteins were available in at least two datasets for meta-analysis, respectively (Supplementary Tables S1 and S2). The results provided evidence for causal associations between two proteins (ALPI [beta = 0.18, SE = 0.04, *p* = 2.02 × 10^−6^], MXRA8 [beta = 0.21, SE = 0.05, *p* = 4.58 × 10^−6^]) and an increased risk of MS, as well as two proteins (IDUA [beta = −0.08, SE = 0.02, *p* = 2.20 × 10^−6^], NID2 [beta = −0.08, SE = 0.02, *p* = 4.53 × 10^−6^]) and a reduced risk of MS (Figure 2). The effect estimates were generally consistent in direction across the different pQTL datasets, although some dataset-specific estimates had wide confidence intervals and did not reach statistical significance individually. The overall associations were supported by the meta-analysis of the four independent pQTL datasets (Figure 3).

For MS severity, two proteins (MANSC1 [beta = 0.09, SE = 0.02, *p* = 6.26 × 10^−6^], LYVE1 [beta = 0.09, SE = 0.02, *p* = 9.51 × 10^−6^]) were associated with greater severity, while one protein CBR1 [beta = −0.08, SE = 0.02, *p* = 7.04 × 10^−6^] showed protective effects (Figure 2 and Figure 3).

Results from sensitivity analyses confirmed the robustness of the primary MR analyses (Supplementary Tables S3 and S4). A few protein-dataset combinations showed evidence of heterogeneity (Cochran’s Q *p* < 0.05) and outlier instruments (MR-PRESSO *p* < 0.05), specifically ALPI and IDUA in the deCODE dataset, and LYVE1 in both Fenland and deCODE datasets. However, in all cases the MR-PRESSO distortion test was non-significant (*p* > 0.05), indicating that the removal of outlier instruments did not materially alter the causal estimates. Furthermore, MR-Egger intercept tests were non-significant across all analyses (*p* > 0.05), providing no evidence of directional pleiotropy. The use of random-effects IVW in the meta-analysis further accounts for residual heterogeneity across instruments. The MR Steiger directionality test consistently supported the hypothesized causal direction from genetically predicted plasma protein levels to MS susceptibility or severity for all significant associations. In addition, all instrumental variables had F statistics exceeding the conventional threshold of 10, indicating sufficient instrument strength and minimizing the risk of weak instrument bias. Collectively, these sensitivity analyses support the robustness of the primary findings.

## 4. Discussion

Our proteome-wide Mendelian randomization analysis provides genetic evidence supporting the involvement of seven plasma proteins in MS susceptibility and severity, highlighting biological pathways related to gut–brain axis signaling, extracellular matrix remodeling, lysosomal function, blood–brain barrier integrity, and oxidative stress. Specifically, we identified four proteins (ALPI, MXRA8, IDUA, and NID2) associated with MS susceptibility and three proteins (MANSC1, LYVE1, and CBR1) associated with MS severity. These findings extend current knowledge beyond genetic susceptibility loci by prioritizing genetically supported protein candidates and biological pathways for future mechanistic investigation, biomarker evaluation, and therapeutic target validation.

Our MR analysis identified four plasma proteins with causal effects on MS susceptibility, implicating novel biological pathways in disease development. The association between elevated ALPI levels and increased MS risk suggests an important role for intestinal barrier function in MS pathogenesis. ALPI is primarily expressed in intestinal epithelial cells and plays an important role in the maintenance of intestinal microbial homeostasis and intestinal barrier function through its ability to dephosphorylate lipopolysaccharide [14]. Recent evidence indicates that gut dysbiosis and increased intestinal permeability may contribute to neuroinflammation through multiple mechanisms, such as molecular mimicry, bystander activation, and systemic immune activation [15]. The gut–brain axis has emerged as a potential modulator of MS severity, with several studies demonstrating that MS patients display an increased prevalence of mucosal inflammation, including alterations in gut microbial composition and intestinal permeability [16,17]. These findings suggest that ALPI-mediated gut barrier dysfunction represents a biologically plausible pathway involved in MS pathogenesis. The association between MXRA8 levels and MS risk implicates extracellular matrix (ECM) remodeling in MS development. MXRA8 is a receptor for multiple arthritogenic alphaviruses, but also functions as a key regulator of cell adhesion and ECM remodeling [18]. In the CNS, the expression of limitrin encoded by the gene *MXRA8* on circulating CD4+ T cells was increased in patients with MS, and expression of limitrin ligands was increased on the blood–brain barrier (BBB) endothelium upon inflammation and in MS lesions [19]. Previous study has shown that ECM components are significantly altered in MS lesions, contributing to both inflammatory demyelination and impaired remyelination [20]. Our findings implicate MXRA8-mediated ECM remodeling in promoting neuroinflammation, likely through facilitating immune cell trafficking across the BBB. *IDUA* (α-L-Iduronidase) is a lysosomal enzyme responsible for glycosaminoglycan (GAG) degradation, whose deficiency causes Mucopolysaccharidosis I, a storage disorder associated with neuroinflammation, microglial activation, and dysregulation of adaptive immunity [21]. Beyond its role in glycosaminoglycan degradation, IDUA might regulate microglial activation and neuroinflammation. While the mechanism has been less studied, evidence has suggested that impaired lysosomal function might contribute to neurological autoimmune diseases including MS [22]. NID2 (Nidogen-2) is a key component of the vascular basement membrane that interacts with laminins and collagen IV to maintain BBB integrity [23]. In MS, BBB disruption is an early event in lesion formation, allowing the entry of immune cells and inflammatory mediators into the CNS [24]. Our results imply that NID2 may protect against MS by stabilizing the BBB and limiting neuroinflammation, suggesting that NID2 may represent a biologically relevant candidate for further investigation in the context of MS. Collectively, these findings highlight distinct but potentially interconnected biological pathways involved in MS susceptibility. Although these proteins represent promising candidates for future biomarker and therapeutic research, independent genetic replication, tissue-specific analyses, and functional studies will be essential to validate their biological relevance and determine their translational potential.

Our findings also reveal evidence for the involvement of LYVE1, CBR1, and MANSC1 in modulating MS disease severity. The association of elevated LYVE1 levels with increased disability provides compelling genetic evidence supporting the involvement of meningeal lymphatic function in MS severity. LYVE1 serves as the primary receptor for hyaluronan on lymphatic endothelium and plays crucial roles in immune cell trafficking and interstitial fluid drainage [25]. Our findings complement recent neuropathological studies suggesting an important role of meningeal lymphatic function in MS [26]. The protective association of CBR1 highlights the importance of oxidative stress management in MS severity. CBR1 plays a central role in controlling redox balance and detoxifying lipid peroxidation, particularly 4-hydroxynonenal (4-HNE) [27], which induces cellular and mitochondrial dysfunction and leads to an impaired endogenous antioxidant response [28]. In active demyelinating MS lesions, 4-HNE accumulates in both phagocytic macrophages and large hypertrophic astrocytes [29]. Therefore, CBR1 might protect against oxidative stress and damage via the reduction in the amount of ROS and reactive toxic aldehyde derivatives that are derived from lipid peroxidation. These findings suggest that CBR1-mediated antioxidant pathways may contribute to resilience against MS severity and warrant further mechanistic investigation. Although direct evidence linking MANSC1 to MS remains limited, emerging findings from other neurodegenerative diseases suggest that MANSC1 may participate in biological processes relevant to neuroinflammation. Integrated bioinformatics analyses have identified *MANSC1* as a candidate gene associated with Parkinson’s disease [30], indicating that it may be involved in molecular pathways shared across neurodegenerative disorders. Collectively, these findings suggest that meningeal lymphatic function, oxidative stress regulation, and neuroimmune pathways may contribute to inter-individual differences in MS severity. However, these observations should be interpreted with caution. Independent genetic replication, tissue-specific analyses, and functional studies are required to confirm the biological relevance of these proteins and to determine whether they represent viable biomarkers or therapeutic targets.

It should be noted that the reported MR effect estimates represent the effect of a one standard deviation genetically predicted increase in standardized plasma protein levels rather than absolute changes in circulating protein concentrations. Because the original pQTL GWAS datasets quantified protein abundance using normalized and standardized measurements, the estimated effects cannot be directly translated into absolute concentration changes. Therefore, although these effect sizes provide genetic evidence supporting protein–MS associations, they should not be interpreted as clinically actionable thresholds. Future prospective studies measuring absolute plasma protein concentrations will be required to determine the clinical relevance and translational potential of these findings.

While our study provides novel insights into the relationships between genetically predicted plasma proteins and MS susceptibility and severity, several limitations should be acknowledged. First, the predominantly European ancestry of the study populations may limit the generalizability of our findings to other ethnic groups, given the known differences in genetic architecture and pQTL effects across populations. Second, although plasma proteins are practical and readily accessible biomarkers, genetically predicted plasma protein levels may not accurately reflect protein abundance within the central nervous system (CNS) or cerebrospinal fluid (CSF). This is particularly relevant in MS because the BBB restricts molecular exchange between the peripheral circulation and the CNS, and the disease is characterized by compartmentalized CNS inflammation. Consequently, the identified plasma proteins should be interpreted as circulating biomarkers or genetically supported candidate therapeutic targets rather than direct surrogates of CNS protein expression. Third, although comprehensive sensitivity analyses were performed, formal colocalization analyses were not conducted. Because the primary analyses integrated both cis- and trans-pQTLs across four independent pQTL datasets, a unified colocalization analysis was not straightforward. Consequently, shared causal variants underlying the protein and MS association signals could not be confirmed, and the possibility that some associations were influenced by linkage disequilibrium between distinct causal variants cannot be completely excluded. Fourth, the MR approach assumes linear relationships between genetically predicted protein levels and disease outcomes and therefore may not capture threshold or non-linear effects. Future studies incorporating multi-ancestry cohorts, integrating plasma, CSF, and brain tissue proteomics, and performing functional validation will be important to further elucidate the biological roles of the identified proteins in MS.

## 5. Conclusions

Our proteome-wide MR analysis provides evidence implicating specific plasma proteins in the susceptibility and severity of MS. These findings expand our understanding of MS pathophysiology beyond established immune mechanisms, revealing novel biological pathways involving gut–brain axis signaling, ECM remodeling, and vascular integrity. The identified proteins represent genetically supported candidate biomarkers and candidate therapeutic targets that warrant further independent validation, tissue-specific analyses, and functional studies before their clinical utility can be established.

## Data Availability

Summary statistics of multiple sclerosis and protein quantitative trait locus could be found in the original publications.

## Supplementary Materials

The following supporting information can be downloaded at: https://www.mdpi.com/article/10.3390/genes17070810/s1, Table S1: Complete proteome-wide Mendelian randomization results for MS susceptibility. Table S2: Complete proteome-wide Mendelian randomization results for MS severity. Table S3: Instrumental variables used for the significant proteins in proteome-wide Mendelian randomization analysis. Table S4: Sensitivity analysis results for the significant proteins.

## Abbreviations

MR	Mendelian randomization
GWAS	genome-wide association study
pQTL	protein quantitative trait locus
IV	instrumental variables
MS	multiple sclerosis
SNP	single nucleotide polymorphisms
ECM	extracellular matrix
BBB	blood–brain barrier
